# The revised recommendation for administering vitamin C in septic patients: The Japanese Clinical Practice Guidelines for Management of Sepsis and Septic Shock 2020

**DOI:** 10.1002/ams2.801

**Published:** 2022-11-22

**Authors:** Guideline Committee of The Japanese Clinical Practice Guidelines for Management of Sepsis and Septic Shock 2020

**Affiliations:** ^1^ Japanese Society of Intensive Care Medicine Tokyo Japan; ^2^ Japanese Association for Acute Medicine Tokyo Japan

To the Editor,

Given the available clinical evidence through the literature search when the Japanese Clinical Practice Guidelines for Management of Sepsis and Septic Shock 2020 (J‐SSCG2020) was creating, J‐SSCG2020^1^, ^2^ suggested administering vitamin C to septic patients based on the 11 available randomized control trials (RCT).^3^, ^4^, ^5^, ^6^, ^7^, ^8^, ^9^, ^10^, ^11^, ^12^, ^13^

Recently, Lamontagne *et al*.^14^ conducted a large multi‐center RCT, including 872 septic patients, who required vasopressors, to evaluate the effect of high‐dose vitamin C. This RCT revealed that the proportion of a composite of death or persistent organ dysfunction at 28 days in the vitamin C group was significantly higher than that in the placebo group. Additionally, several RCTs were published after our meta‐analysis on this issue for J‐SSCG2020. Therefore, we performed an updated systematic review on 20th June 2022. We identified 12 new RCTs^14^, ^15^, ^16^, ^17^, ^18^, ^19^, ^20^, ^21^, ^22^, ^23^, ^24^, ^25^ and performed an updated meta‐analysis using these 23 RCTs (Table 1 and Appendix S1).

**Table 1:** Evidence profile

Certainty assessment							№ of patients		Effect		Certainty	Importance
№ of studies	Study design	Risk of bias	Inconsistency	Indirectness	Imprecision	Other considerations	Vitamin C	Placebo	Relative (95% CI)	Absolute (95% CI)		
Long term mortality (more than 60 days)												
6	Randomized trials	Serious^†^	Not serious	Not serious	Not serious	None	607/1,440 (42.2%)	545/1,441 (37.8%)	**RR 1.11** (1.02 to 1.22)	**42 more per 1,000** (from 8 more to 83 more)	⨁⨁⨁◯ Moderate	CRITICAL
28 or 30 days mortality												
15	Randomized trials	Very serious^‡^	Serious^§^	Not serious	Not serious	None	573/1,940 (29.5%)	584/1,916 (30.5%)	**RR 0.89** (0.77 to 1.04)	**34 fewer per 1,000** (from 70 fewer to 12 more)	⨁◯◯◯Very low	CRITICAL
In‐hospital mortality												
12	Randomized trials	Very serious^‡^	Serious^¶^	Not serious	Not serious	None	383/1,194 (32.1%)	382/1,150 (33.2%)	**RR 0.94** (0.76 to 1.16)	**20 fewer per 1,000** (from 80 fewer to 53 more)	⨁◯◯◯Very low	CRITICAL
Length of ICU stay (days)												
16	Randomized trials	Very serious^‡^	Very serious^††^	Not serious	Not serious	NONE	1,785	1,749	‐	MD **0.25 lower** (0.72 lower to 0.22 higher)	⨁◯◯◯Very low	CRITICAL
Length of hospital stay (days)												
12	Randomized trials	Very serious^‡^	Very serious^‡‡^	Not serious	Not serious	None	1,722	1,685	‐	MD **0.24 higher** (0.97 lower to 1.45 higher)	⨁◯◯◯Very low	CRITICAL
Acute kidney injury												
9	Randomized trials	Very serious^‡^	Not serious	Not serious	Not serious	None	338/1,113 (30.4%)	324/1,117 (29.0%)	**RR 1.02** (0.93 to 1.13)	**6 more per 1,000** (from 20 fewer to 38 more)	⨁⨁◯◯Low	CRITICAL

CI, confidence interval; MD, mean difference; RR, risk ratio.

^†^ One study with a high risk of bias was included.

^‡^ Two or more studies with high risk of bias were included.

^§^ The *I* ^2^ value was 39%.

^¶^ The *I* ^2^ value was 50%.

^††^ The *I* ^2^ value was 68%. We thought that this value is quite large.

^‡‡^ The *I* ^2^ value was 70%. We thought that this value is quite large.

In our updated meta‐analysis, the estimated value of the desirable anticipated effect was as follows: the length of ICU stay yielded a mean difference (MD) of 0.25 days shorter (95% Confidence interval (CI): 0.72 days shorter–0.22 days longer) (16 RCTs, *n* = 3,534). Thereby, the desirable anticipated effect was thought to be “trivial.” The estimated values of the effects on mortality were as follows: long‐term mortality, namely more than 60 days, yielded a risk difference (RD) of 42 more per 1,000 (95% CI: 8 more–83 more; 6 RCTs, *n* = 2,881), 28 or 30 days mortality yielded an RD of 34 fewer per 1,000 (95% CI: 70 fewer–12 more; 15 RCTs, *n* = 3,856), in‐hospital mortality yielded an RD of 20 fewer per 1,000 (95% CI: 80 fewer–53 more; 12 RCTs, *n* = 2,344). Of these three mortalities, long‐term mortality was chosen as the effect on mortality since we predetermined that the highest certainty of evidence was adopted. Subsequently, the estimated values of the other undesirable anticipated effects were as follows: the length of hospital stay yielded an MD of 0.24 days longer (95% CI: 0.97 days shorter–1.45 days longer; 12 RCTs, *n* = 3,407), and acute kidney injury yielded an RD of 6 more per 1,000 (95% CI: 20 fewer–38 more; 9 RCTs, *n* = 2,230). Thereby, the undesirable anticipated effects were “moderate.” Thus, we presumed that administering vitamin C was inferior to the placebo or control. Judgment of values, acceptability, and feasibility were not changed, namely, “probably no important uncertainty or variability,” “probably yes,” and “probably yes,” respectively.

Accordingly, we revised our recommendation to “We suggest against administering vitamin C to septic patients (GRADE 2D: certainty of evidence = “very low”).”

## DECLARATIONS

Ethics approval and consent to participate: Not applicable.

Consent for publication: Not applicable.

## DISCLOSURE

Conflicts of Interest (COIs) and members' roles: All financial and non‐financial competing interests were declared in the J‐SSCG2020.^1^, ^2^

## Supporting information


**Appendix S1.** PRISMA flow diagram, risk of bias summary, forrest plot, funnel plot, and evidence to decision table.

## Data Availability

All data generated or analyzed during this study are included in this published article and its Appendix S1 files.
