# Supplementary material for: The revised recommendation for administering vitamin C in septic patients: The Japanese Clinical Practice Guidelines for Management of Sepsis and Septic Shock 2020
Source: Acute Med Surg. 2022 Nov 22;9(1):e801. doi: 10.1002/ams2.801 (PMC9679883; doi:10.1002/ams2.801)
Supplement: Supplementary file 1 — Appendix S1. PRISMA flow diagram, risk of bias summary, forrest plot, funnel plot, and evidence to decision table. [file AMS2-9-e801-s001.pdf]

Additional File

PRISMA flow diagram

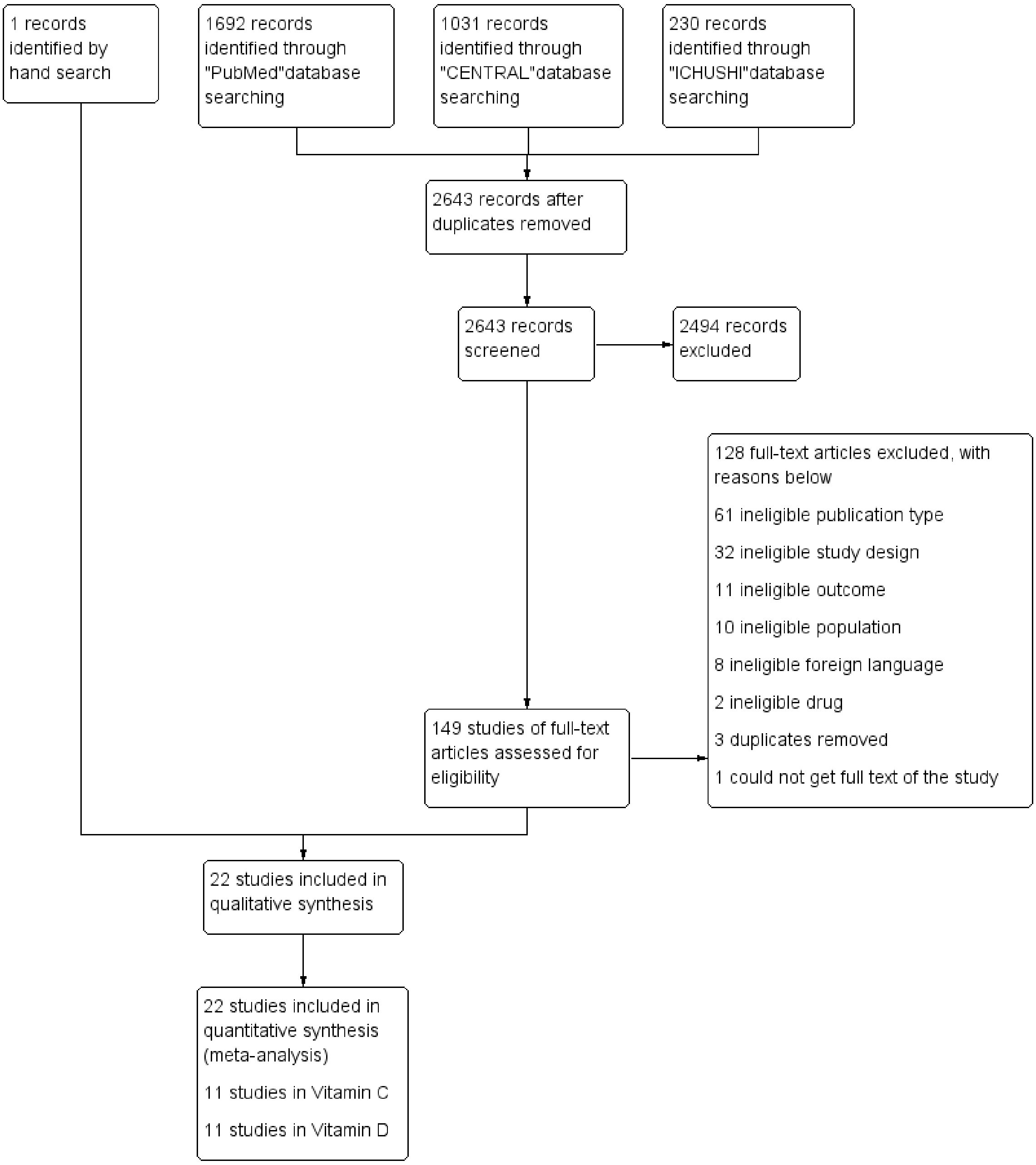

Additional systematic review

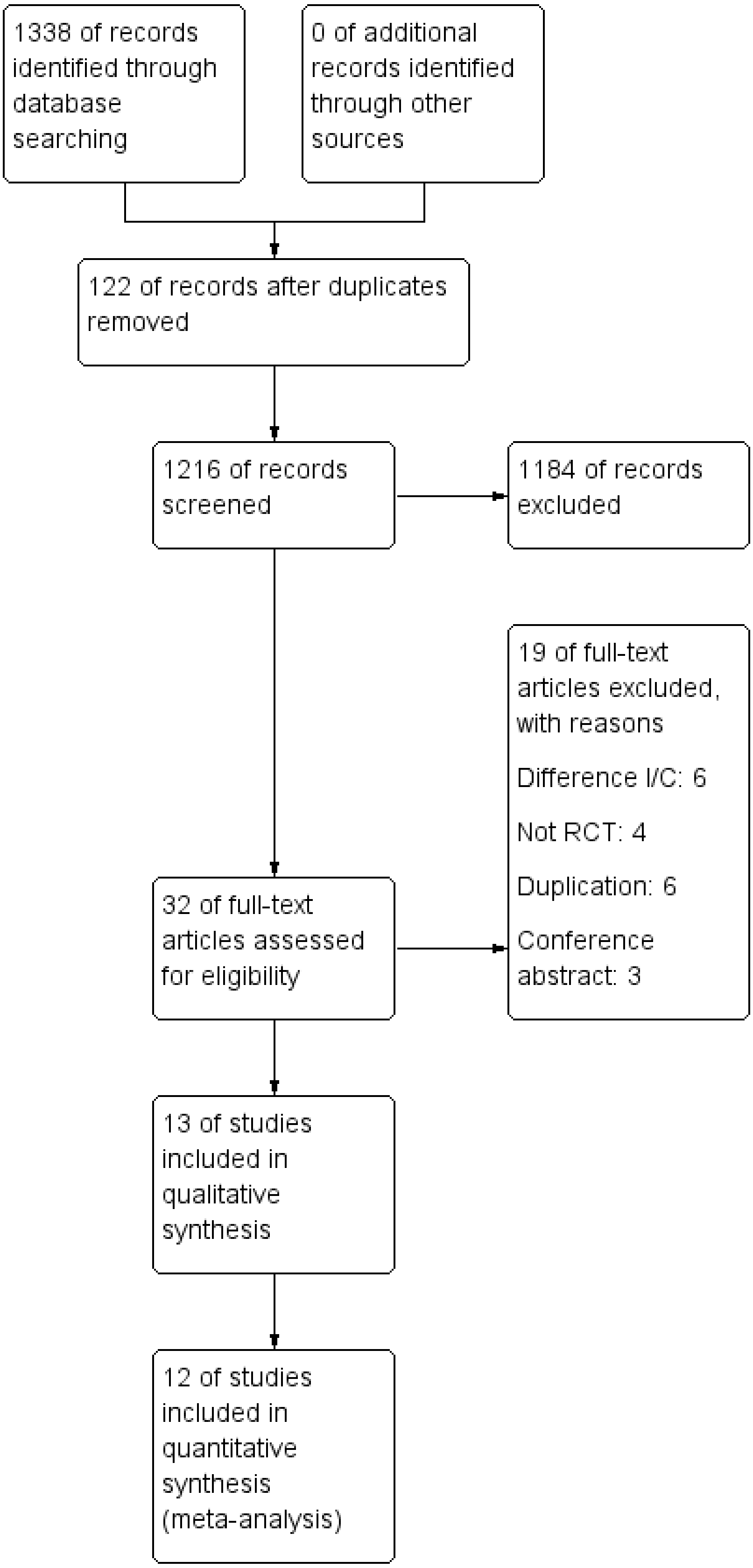

Risk of Bias summary

	Random sequence generation (selection bias)	Allocation concealment (selection bias)	Blinding of participants and personnel (performance bias)	Blinding of outcome assessment (detection bias)	Incomplete outcome data (attrition bias)	Selective reporting (reporting bias)	Other bias
Crimi 2004	+	+	+	+	+	+	+
Ferron-Celma 2009	+	+	?	?	+	-	+
Fowler 2014	+	+	+	+	+	+	+
Fowler 2019	+	+	+	+	+	+	+
Fujii 2020	+	+	+	+	+	+	+
Heyland 2013	+	+	+	+	+	+	+
Howe 2015	+	+	+	+	+	-	+
Hussein 2021	+	-	-	+	+	+	+
Hwang SY, 2020	+	+	+	+	+	+	+
Iglesias J 2020	+	+	+	+	+	+	+
Jamshidi 2021	+	+	-	+	+	+	+
Lamontagne 2022	+	+	+	+	+	+	+
Mahmoodpoor A 2021	+	+	+	+	+	+	+
Moskowitz A 2020	+	+	+	+	+	+	+
Nogueira 2013	?	?	+	+	-	-	+
Ping Chang 2020	+	+	-	+	+	+	+
Porter 1999	+	+	+	+	+	-	+
Rosengrave P 2022	+	+	+	+	+	+	+
Sevransky JE 2021	+	+	+	+	+	+	-
Shi-Jin Lv 2021	+	-	-	+	+	-	+
Tanaka 2000	-	-	+	+	+	-	?
Wacker DA 2022	+	+	+	+	+	+	+
Zabet 2016	+	+	+	+	+	+	+

Forrest plot

Outcome ① Long term mortality（more than 60 days）

Study or Subgroup	Vitamin C Events	Vitamin C Total	Placebo Events	Placebo Total	Weight	Risk Ratio M-H, Random, 95% CI
Fujii 2020	30	105	25	102	3.8%	1.17 [0.74, 1.84]
Heyland 2013	259	593	218	586	40.9%	1.17 [1.02, 1.35]
Hwang SY, 2020	17	53	16	58	2.4%	1.16 [0.66, 2.06]
Lamontagne 2022	191	417	185	426	35.1%	1.05 [0.91, 1.23]
Rosengrave P 2022	8	20	7	20	1.2%	1.14 [0.51, 2.55]
Sevransky JE 2021	102	252	94	249	16.6%	1.07 [0.86, 1.33]
**Total (95% CI)**		**1440**		**1441**	**100.0%**	**1.11 [1.02, 1.22]**
Total events	607		545			

Heterogeneity: Tau² = 0.00; Chi² = 1.23, df = 5 (P = 0.94); I² = 0%
Test for overall effect: Z = 2.35 (P = 0.02)

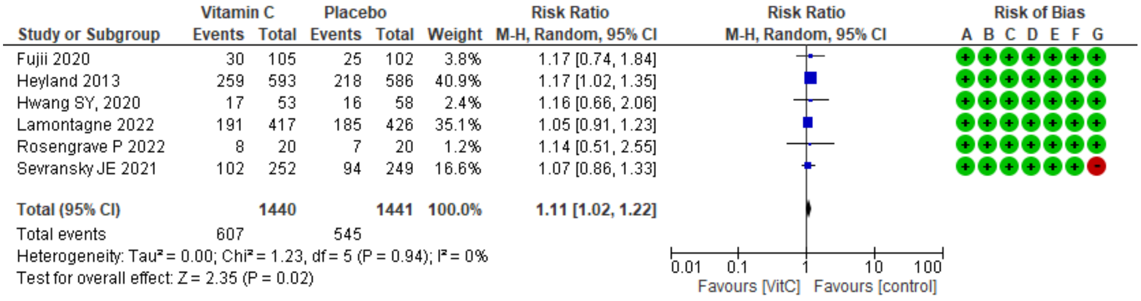

Outcome ② 28 or 30 days mortality

Study or Subgroup	Vitamin C Events	Vitamin C Total	Placebo Events	Placebo Total	Weight	Risk Ratio M-H, Random, 95% CI
Fowler 2014	7	16	5	8	3.2%	0.70 [0.32, 1.52]
Fowler 2019	25	84	38	83	8.3%	0.65 [0.43, 0.97]
Fujii 2020	24	106	21	103	5.9%	1.11 [0.66, 1.87]
Heyland 2013	190	617	173	601	16.6%	1.07 [0.90, 1.27]
Hussein 2021	17	47	21	47	6.3%	0.81 [0.49, 1.33]
Hwang SY, 2020	11	53	9	58	3.0%	1.34 [0.60, 2.97]
Lamontagne 2022	152	429	137	434	15.9%	1.12 [0.93, 1.36]
Mahmoodpoor A 2021	6	40	11	40	2.5%	0.55 [0.22, 1.33]
Moskowitz A 2020	35	101	29	99	8.2%	1.18 [0.79, 1.78]
Ping Chang 2020	11	40	14	40	4.1%	0.79 [0.41, 1.52]
Rosengrave P 2022	6	20	7	20	2.4%	0.86 [0.35, 2.10]
Sevransky JE 2021	56	252	60	249	10.8%	0.92 [0.67, 1.27]
Shi-Jin Lv 2021	15	61	24	56	5.7%	0.57 [0.34, 0.98]
Wacker DA 2022	16	60	26	64	6.0%	0.66 [0.39, 1.10]
Zabet 2016	2	14	9	14	1.2%	0.22 [0.06, 0.85]
**Total (95% CI)**		**1940**		**1916**	**100.0%**	**0.89 [0.77, 1.04]**
Total events	573		584			

Heterogeneity: Tau² = 0.03; Chi² = 23.06, df = 14 (P = 0.06); I² = 39%
Test for overall effect: Z = 1.50 (P = 0.13)

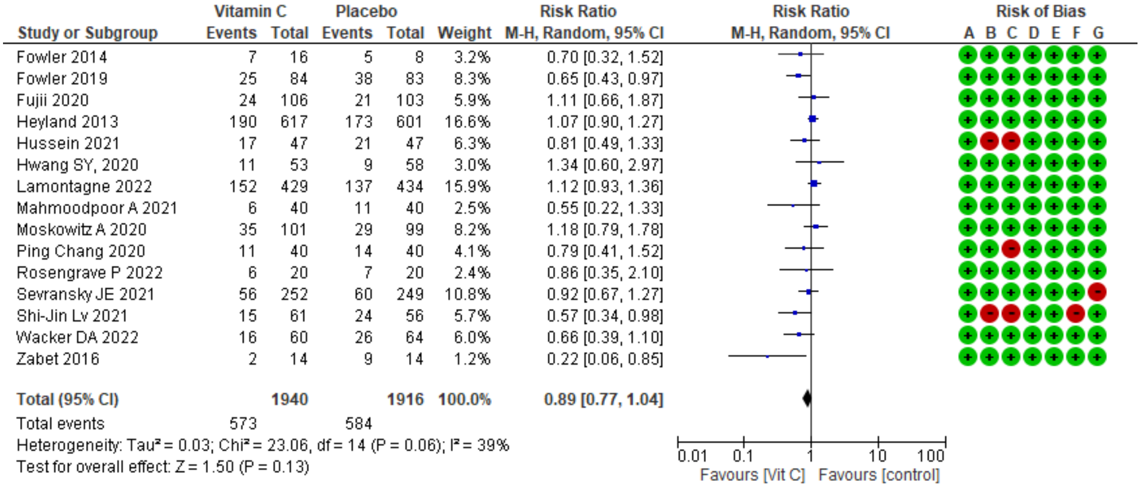

Outcome ③ In-hospital mortality

Study or Subgroup	Vitamin C Events	Vitamin C Total	Placebo Events	Placebo Total	Weight	Risk Ratio M-H, Random, 95% CI
Crimi 2004	49	112	76	112	18.2%	0.64 [0.50, 0.82]
Ferron-Celma 2009	6	10	4	10	4.5%	1.50 [0.60, 3.74]
Fujii 2020	24	106	21	103	9.9%	1.11 [0.66, 1.87]
Heyland 2013	216	617	199	601	21.2%	1.06 [0.90, 1.24]
Howe 2015	17	50	10	22	8.3%	0.75 [0.41, 1.36]
Hwang SY, 2020	13	53	11	58	6.6%	1.29 [0.63, 2.63]
Iglesias J 2020	11	68	13	69	6.3%	0.86 [0.41, 1.78]
Jamshidi 2021	3	29	11	29	2.9%	0.27 [0.08, 0.88]
Moskowitz A 2020	28	101	23	99	10.9%	1.19 [0.74, 1.92]
Porter 1999	0	9	0	9		Not estimable
Rosengrave P 2022	7	20	7	20	5.1%	1.00 [0.43, 2.33]
Tanaka 2000	9	19	7	18	6.1%	1.22 [0.58, 2.57]
**Total (95% CI)**		**1194**		**1150**	**100.0%**	**0.94 [0.76, 1.16]**
Total events	383		382			

Heterogeneity: Tau² = 0.05; Chi² = 19.87, df = 10 (P = 0.03); I² = 50%
Test for overall effect: Z = 0.58 (P = 0.56)

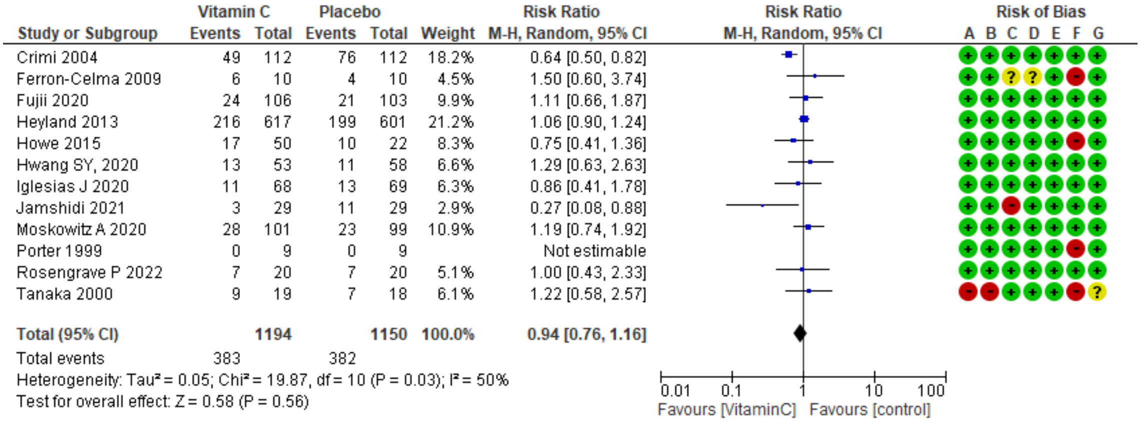

Outcome ④ Length of ICU stay

Study or Subgroup	Vitamin C Mean	Vitamin C SD	Vitamin C Total	Placebo Mean	Placebo SD	Placebo Total	Weight	Mean Difference IV, Random, 95% CI
Fowler 2014	8.6	6.7841645	16	11	8.0184066	8	0.5%	-2.40 [-8.87, 4.07]
Heyland 2013	9.4333333	7.9506918	617	9.9333333	7.9511923	601	10.7%	-0.50 [-1.39, 0.39]
Howe 2015	12.946	9.718924	50	19.1	16	22	0.4%	-6.15 [-13.36, 1.05]
Hussein 2021	8.319	4.071	47	9.787	4.206	47	5.5%	-1.47 [-3.14, 0.21]
Hwang SY, 2020	6.3333333	6.0963038	53	7.3333333	6.4618827	58	3.3%	-1.00 [-3.34, 1.34]
Iglesias J 2020	4.76	4.3	68	4.66	3.45	69	7.5%	0.10 [-1.21, 1.41]
Lamontagne 2022	7	6.6944307	429	6.6666667	5.9504181	433	11.2%	0.33 [-0.51, 1.18]
Mahmoodpoor A 2021	12.77	3.71	40	14.15	3.12	40	8.3%	-1.38 [-2.88, 0.12]
Nogueira 2013	30	11	11	27	13	23	0.3%	3.00 [-5.40, 11.40]
Ping Chang 2020	8.1	6.7665831	40	7.7666667	5.9976532	40	2.4%	0.33 [-2.47, 3.14]
Porter 1999	22	25.2	9	35.8	24.3	9	0.0%	-13.80 [-36.67, 9.07]
Rosengrave P 2022	4.9	1.9	20	9.3	4.2	20	4.2%	-4.40 [-6.42, -2.38]
Sevransky JE 2021	4.5	1	250	4.5	1	245	17.0%	0.00 [-0.18, 0.18]
Shi-Jin Lv 2021	4.9	1.3	61	4.6	1.1	56	15.2%	0.30 [-0.14, 0.74]
Wacker DA 2022	3.8	1.4	60	3	1	64	15.2%	0.80 [0.37, 1.23]
Zabet 2016	21.45	10.23	14	20.57	13.04	14	0.3%	0.88 [-7.80, 9.56]
Total (95% CI)			1785			1749	100.0%	-0.25 [-0.72, 0.22]

Heterogeneity: Tau² = 0.34; Chi² = 46.46, df = 15 (P < 0.0001); I² = 68%
Test for overall effect: Z = 1.04 (P = 0.30)

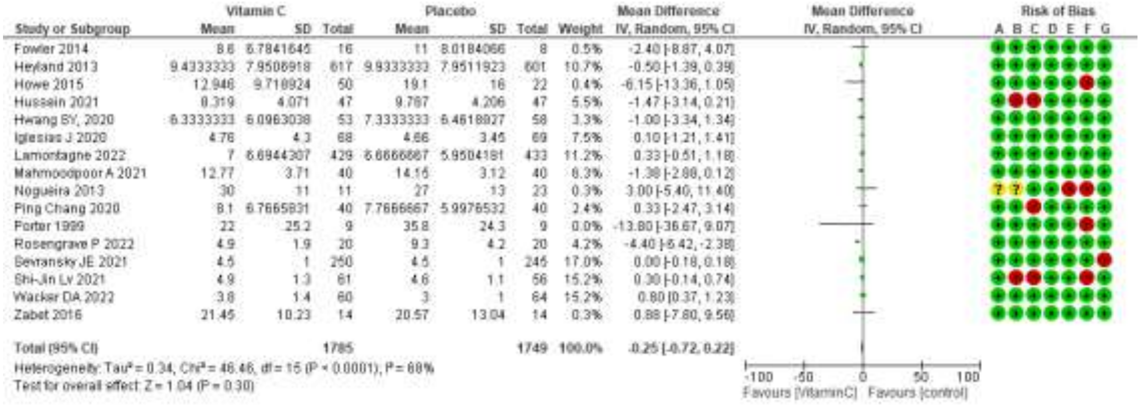

Outcome ⑤ Length of hospital stay

Study or Subgroup	Vitamin C Mean	Vitamin C SD	Vitamin C Total	Placebo Mean	Placebo SD	Placebo Total	Weight	Mean Difference IV, Random, 95% CI
Fujii 2020	14.83333333	14.87941847	107	14.866667	14.960461	104	6.1%	-0.03 [-4.06, 3.99]
Heyland 2013	20.366667	20.95416	617	19.233333	18.503242	601	11.5%	1.13 [-1.09, 3.35]
Howe 2015	22.434	17.391263	50	22.6	15.5	22	2.0%	-0.17 [-8.24, 7.91]
Hussein 2021	9.447	4.226	47	11.17	5.036	47	12.9%	-1.72 [-3.60, 0.16]
Hwang SY, 2020	15.333333	7.6525543	46	16.166667	12.961542	52	5.8%	-0.83 [-4.99, 3.33]
Iglesias J 2020	11.5	6.8	68	11	6.2	69	11.7%	0.50 [-1.68, 2.68]
Lamontagne 2022	18.333333	17.851815	429	18.666667	17.851254	433	10.9%	-0.33 [-2.72, 2.05]
Porter 1999	31.3	23.4	9	49	30	9	0.2%	-17.70 [-42.56, 7.16]
Rosengrave P 2022	20.5	6	20	26.5	10.5	20	4.1%	-6.00 [-11.30, -0.70]
Sevransky JE 2021	10.75	1.8	250	10	2	246	18.5%	0.75 [0.41, 1.09]
Tanaka 2000	40	28	19	49	44	18	0.3%	-9.00 [-32.91, 14.91]
Wacker DA 2022	10.5	4	60	7.2	2.2	64	16.1%	3.30 [2.15, 4.45]
Total (95% CI)			1722			1685	100.0%	0.24 [-0.97, 1.45]

Heterogeneity: Tau² = 2.03; Chi² = 36.20, df = 11 (P = 0.0002); I² = 70%
Test for overall effect: Z = 0.38 (P = 0.70)

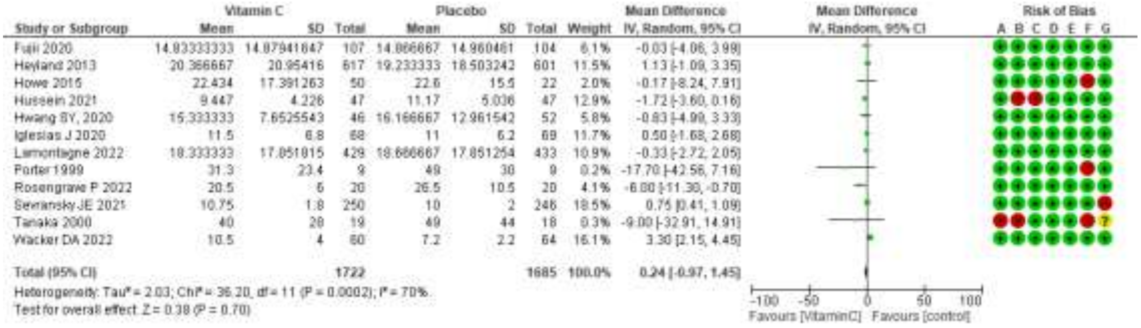

Outcome ⑥ Acute Kidney Injury

Study or Subgroup	Vitamin C Events	Vitamin C Total	Placebo Events	Placebo Total	Weight	Risk Ratio M-H, Random, 95% CI
Fujii 2020	75	107	75	104	32.2%	0.97 [0.82, 1.15]
Hwang SY, 2020	3	37	2	45	0.3%	1.82 [0.32, 10.35]
Iglesias J 2020	54	68	52	69	29.0%	1.05 [0.88, 1.26]
Lamontagne 2022	162	429	164	433	32.6%	1.00 [0.84, 1.18]
Moskowitz A 2020	32	101	27	99	5.1%	1.16 [0.76, 1.79]
Ping Chang 2020	1	40	2	40	0.2%	0.50 [0.05, 5.30]
Sevransky JE 2021	1	252	0	249	0.1%	2.96 [0.12, 72.42]
Tanaka 2000	0	19	0	18		Not estimable
Wacker DA 2022	10	60	2	60	0.4%	5.00 [1.14, 21.86]
**Total (95% CI)**		**1113**		**1117**	**100.0%**	**1.02 [0.93, 1.13]**
Total events	338		324			

Heterogeneity: Tau² = 0.00; Chi² = 6.69, df = 7 (P = 0.46); I² = 0%
Test for overall effect: Z = 0.43 (P = 0.67)

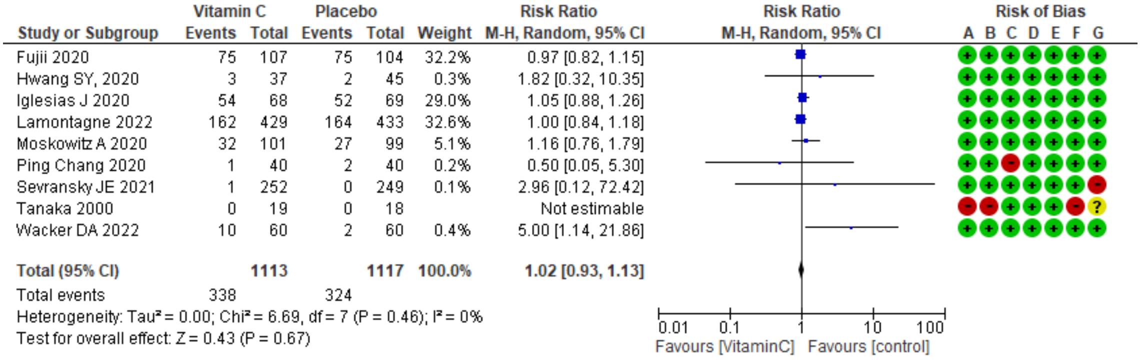

Funnel plot

Outcome ① Long term mortality（more than 60days）

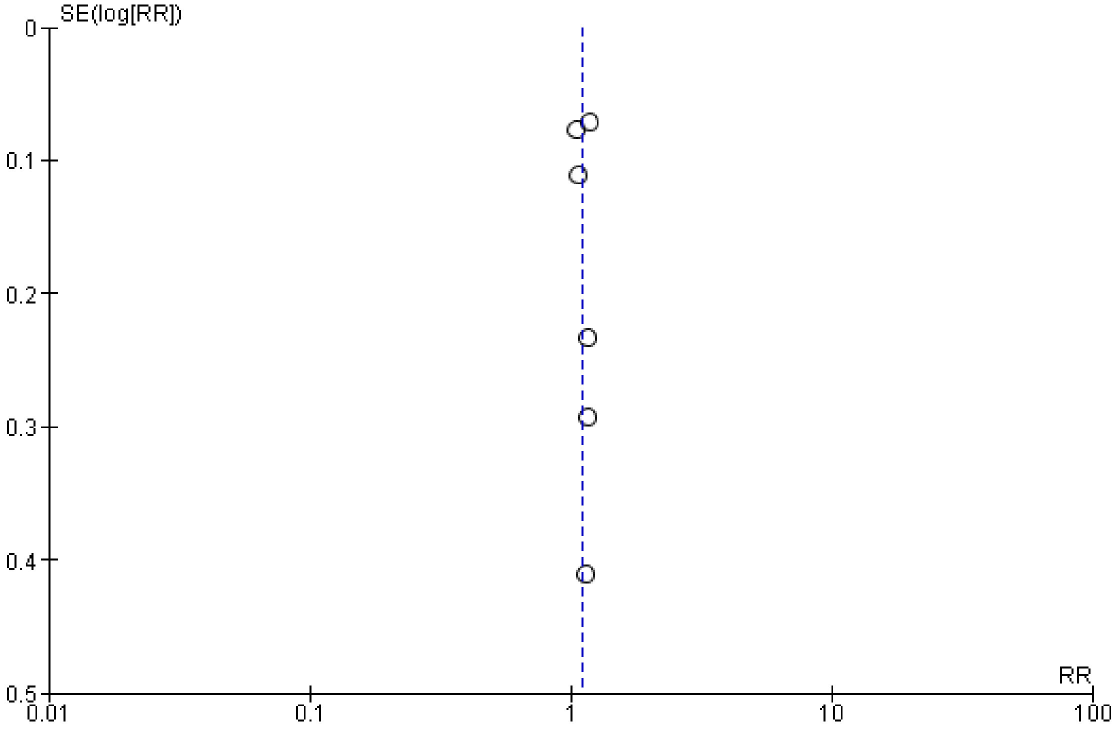

Outcome ② 28 or 30 days mortality

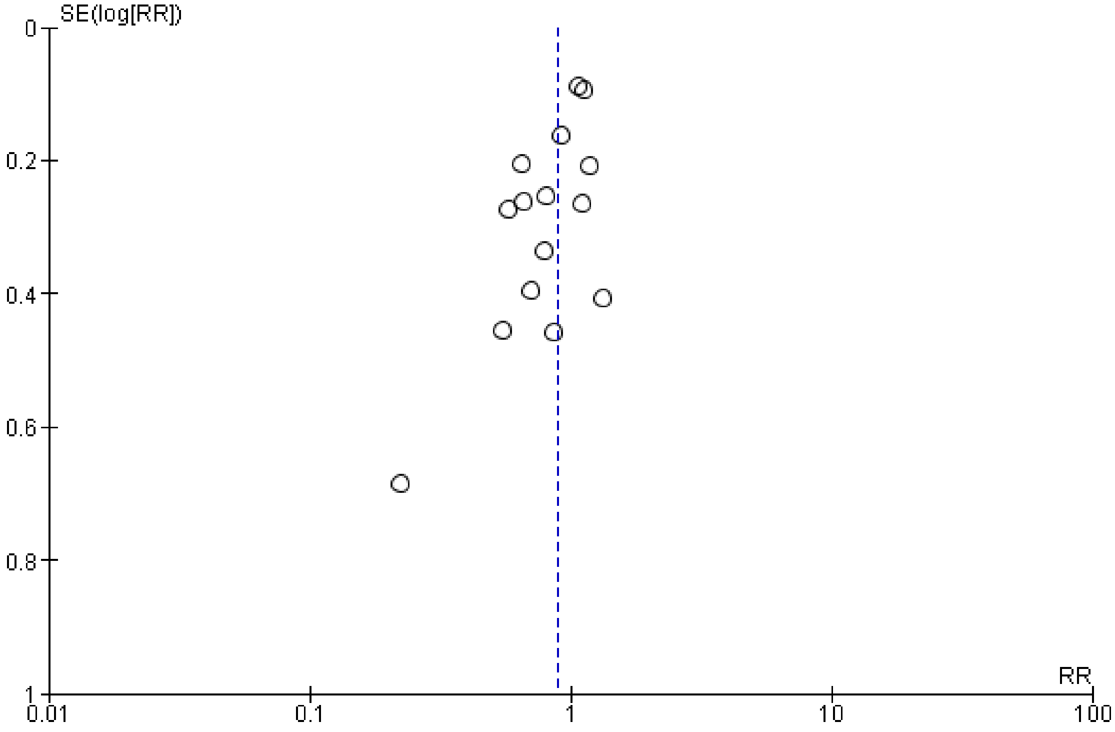

Outcome ③ In-hospital mortality

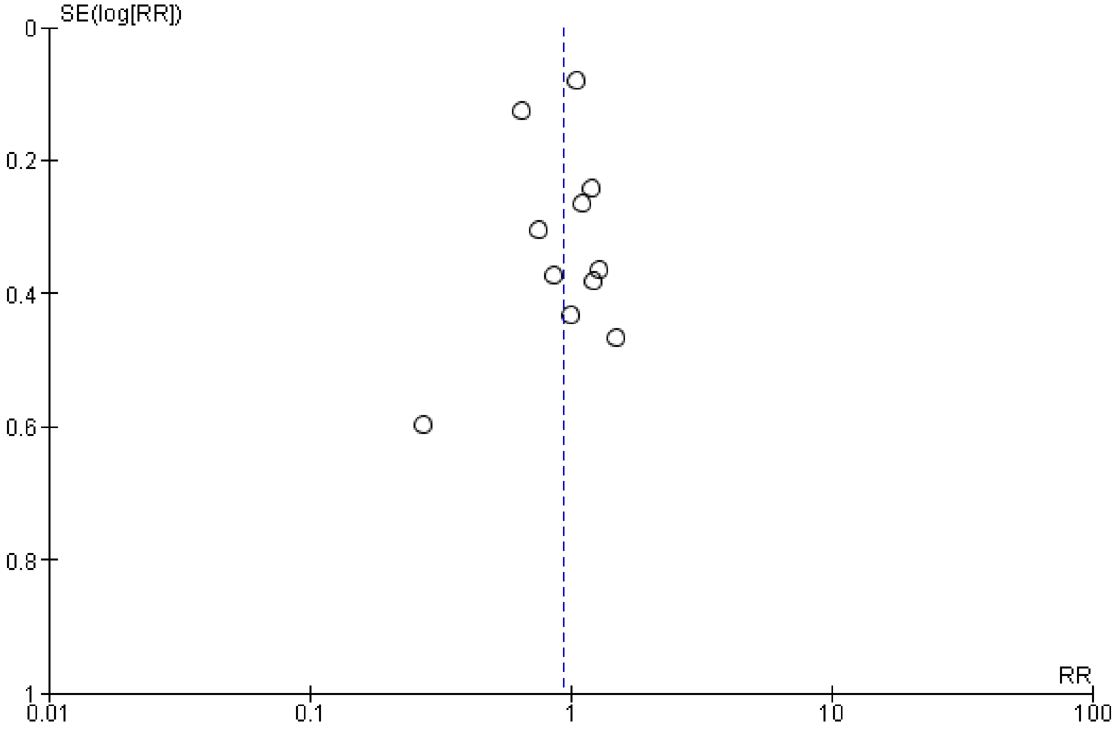

Outcome ④ Length of ICU stay

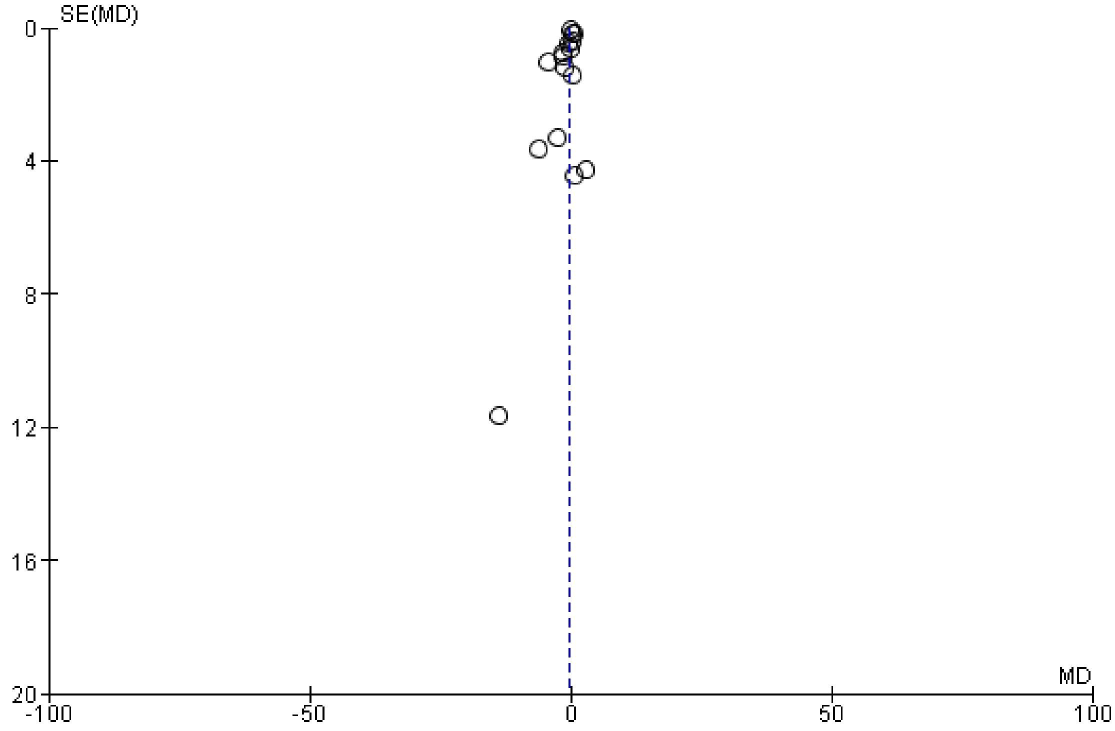

Outcome ⑤　Length of hospital stay

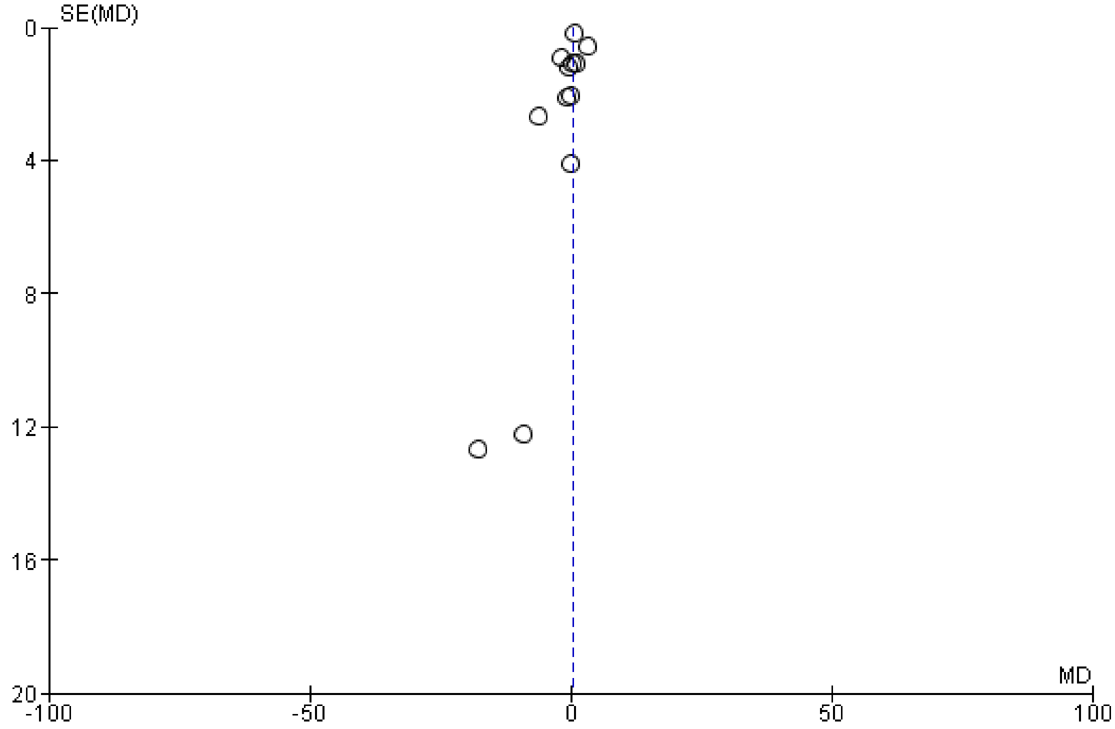

Outcome ⑥　Acute Kidney Injury

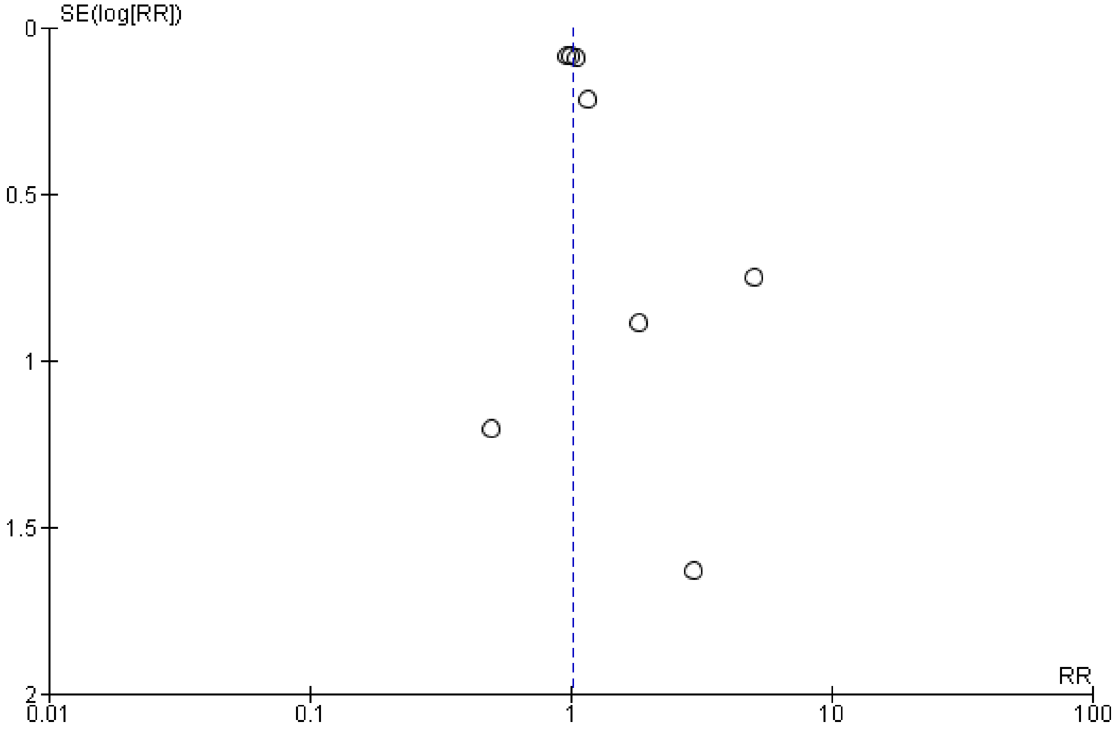

Evidence to Decision Table

	JUDGEMENT						
PROBLEM	No	Probably no	**Probably yes**	Yes		Varies	Don't know
DESIRABLE EFFECTS	**Trivial**	Small	Moderate	Large		Varies	Don't know
UNDESIRABLE EFFECTS	Large	**Moderate**	Small	Trivial		Varies	Don't know
CERTAINTY OF EVIDENCE	**Very low**	Low	Moderate	High			No included studies
VALUES	Important uncertainty or variability	Possibly important uncertainty or variability	**Probably no important uncertainty or variability**	No important uncertainty or variability			
BALANCE OF EFFECTS	Favors the comparison	**Probably favors the comparison**	Does not favor either the intervention or the comparison	Probably favors the intervention	Favors the intervention	Varies	Don't know
ACCEPTABILITY	No	Probably no	**Probably yes**	Yes		Varies	Don't know
FEASIBILITY	No	Probably no	**Probably yes**	Yes		Varies	Don't know
